# A case of sclerochoroidal calcification masquerading as a retained intraocular foreign body

**DOI:** 10.1016/j.radcr.2023.02.049

**Published:** 2023-03-25

**Authors:** Xheida Mani, Richard Johnson

**Affiliations:** a School of Medicine, University of Auckland, Auckland, Aotearoa, 85 Park Road, Grafton, Auckland 1023, New Zealand; b Greenlane Clinical Centre, Auckland, Aotearoa, New Zealand

**Keywords:** Sclerochoroidal calcification, Pseudo intraocular foreign body, Ocular trauma, Ocular computerized tomography

## Abstract

A 41-year-old, New Zealand European male, was seen in the acute eye clinic as an in-patient from the trauma ward with multiple comorbidities including an orbital fracture from injuries sustained in a road traffic accident. It is not uncommon in emergency settings for emergency physicians to review polytrauma patients prior to Ophthalmologists, with Computerized Tomography being the imaging modality of choice. A hyper-dense lesion within the right globe was noted by radiology at the time and concerns were raised about the possibility of a retained intraocular foreign body. Upon Ophthalmic examination, a clinical diagnosis of sclerochoroidal calcification was made. This case highlights a rare case of sclerochoroidal calcification manifesting as a hyperdense lesion on computerized tomography masquerading as an intraocular foreign body.

## Introduction

Globally it is estimated that 55 million eye injuries occur annually [1], of which New Zealand reports an annual eye injury incidence of 1007/1,000,000 population/year [2]. Intraocular foreign bodies are a rare yet serious outcome of ocular injuries with a retrospective population study in Waikato New Zealand reporting a 2.3% incidence of intraocular foreign bodies [3]. Intraocular foreign bodies are usually due to high velocity trauma and can result in penetrating or perforating injuries causing direct mechanical or metallotic damage to surrounding structures [4]. Due to the acute and devastating nature of such injury, a retained intraocular foreign body is considered a surgical emergency requiring an immediate diagnosis and management plan [5].

Radiographic evaluation in the assessment of intraocular foreign bodies is essential, as often patients may present with polytrauma, and history or physical examination can often be limited by the severity of the injuries. Magnetic resonance imaging of any metallic foreign body is contraindicated due to the magnetic field generated causing any metallic material to become dislodged, damaging ocular structures along its trajectory [6]. Computerized tomography (CT) is usually widely available in emergency settings and is used to provide safe, rapid, and detailed evaluation of the osseous and soft tissue structures of the orbit [7]. In patients that have sustained severe head and face trauma, it is not uncommon for radiologists to interpret trauma CT imaging long before a detailed ophthalmic evaluation can be performed [7]. As nearly all metallic foreign bodies (bar aluminium) exhibit as hyperdense areas on CT, the challenge lies with radiologists when similar yet nonvision threatening conditions appear to masquerade as metallic foreign bodies.

This case highlights sclerochoroidal calcification, an idiopathic and usually benign ocular condition that masqueraded as an intraocular foreign body on CT imaging of a patient presenting with polytrauma.

## Case presentation

A 41-year-old, New Zealand European male, was seen in the acute eye clinic as an in-patient from the trauma ward with multiple comorbidities sustained from a car vs bicycle collision. the patient ejected off their bicycle and sustained a multitude of injuries relating to multiorgan perforations, lacerations, and fractures. Most notably, a right tripod facial fracture was detected with concerns raised as to the integrity of the globe and its structures.

## Ocular examination

**Fig. 1:**
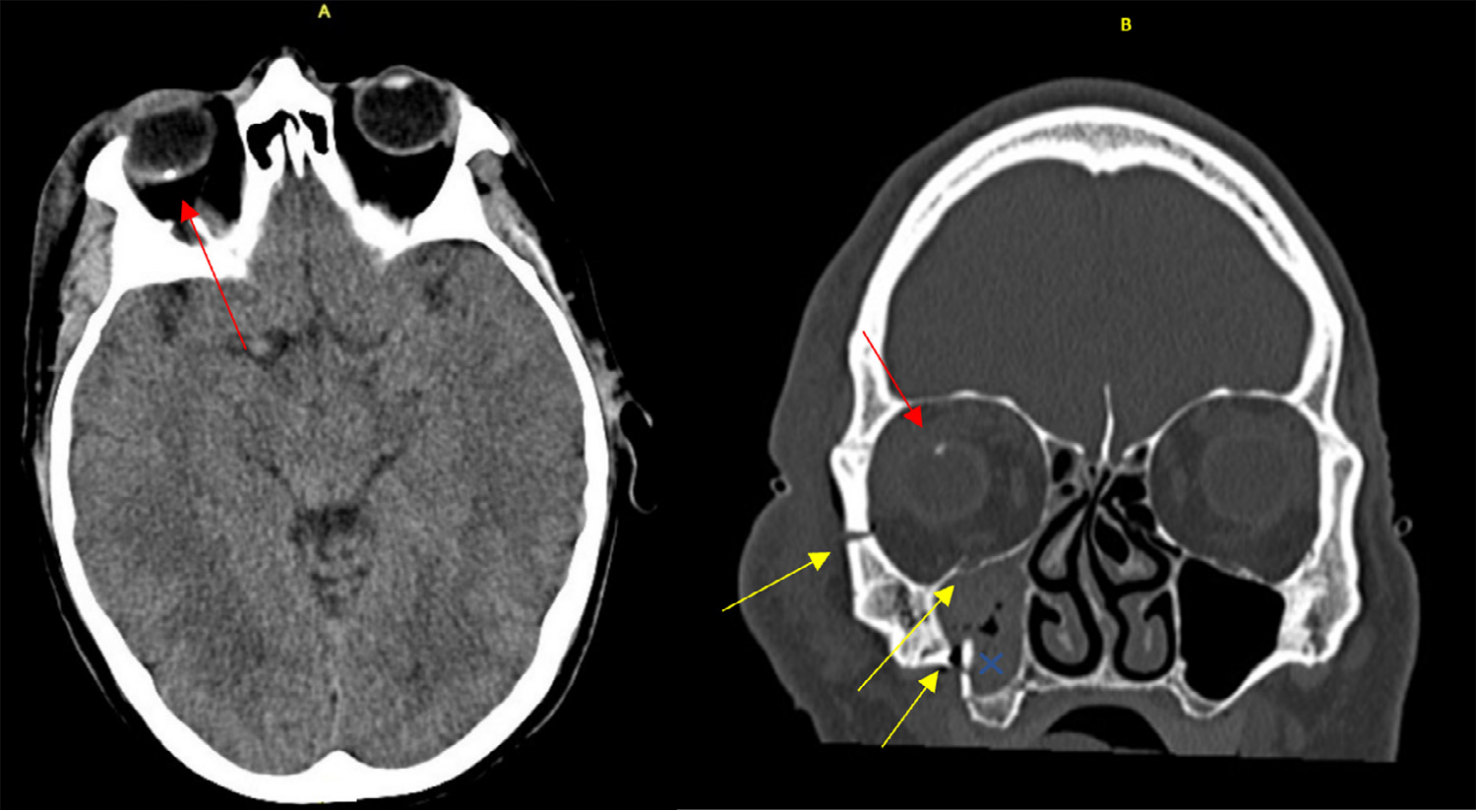
(A) Axial CT showing a hyperdense lesion in the right eye (red arrow). (B) CT scan of his face, which was done at the time of the accident, showed a right sided tripod fracture involving the right orbital floor, zygomatic arch and maxillary sinuses (yellow arrows). There is blood in the right maxillary sinus (x). A hyper-dense lesion is noted in the right eye on CT which raises the possibility of a retained IOFB (red arrow).

Once the patient was stabilized, follow up examinations for the tripod fracture were arranged by the Maxillofacial department with Ophthalmology input requested to investigate the possibility of a retained IOFB, given the hyperattenuating lesion noted in the right eye on CT imaging (Fig. 1).

Background ocular history includes bilateral keratoconus, with a previous 3 right penetrating keratoplasties, with moderate keratoconus remaining in the left eye, predominantly managed with a rigid gas permeable lens.

Unaided visual acuity of the right eye was counting fingers improving to 6/15 with pinhole, left eye aided visual acuity was 6/7.5 with a rigid contact lens in-situ. Intraocular pressures were RE 10 mmHg and LE 6 mmHg.

Eye movements were full and unrestricted in both eyes.  Color plate testing with Ishihara was full and brisk in each eye. Monocular confrontational visual field testing was clear of obvious abnormality in either eye. Pupils were equal and reactive to light with no sign of relative afferent pupillary defect. Exophthalmometry was R 15 mm—112 mm—L 16 mm.

**Fig. 2:**
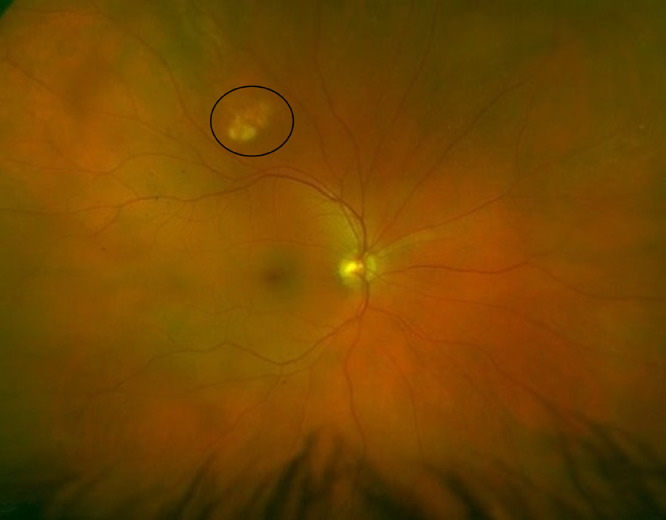
Optos image of the patient's right fundus. Highlighted is a white, raised retinal lesion superotemporal to the right optic nerve head.

Slit lamp examination showed an intact right globe. The right corneal graft was reasonably clear with no sign of rejection or dehiscence, whilst the left cornea showed signs of moderate keratoconus. Both anterior chambers were deep and quiet. The crystalline lens was clear in both eyes, and the vitreous was quiet. Dilated fundus examination showed no abnormality to the left eye, however a raised white retinal lesion was noted in the right eye, superior-temporal to the optic disc approximately 4-disc diameters away and approximately 1.5-disc diameters in length (Fig. 2)

**Fig. 3:**
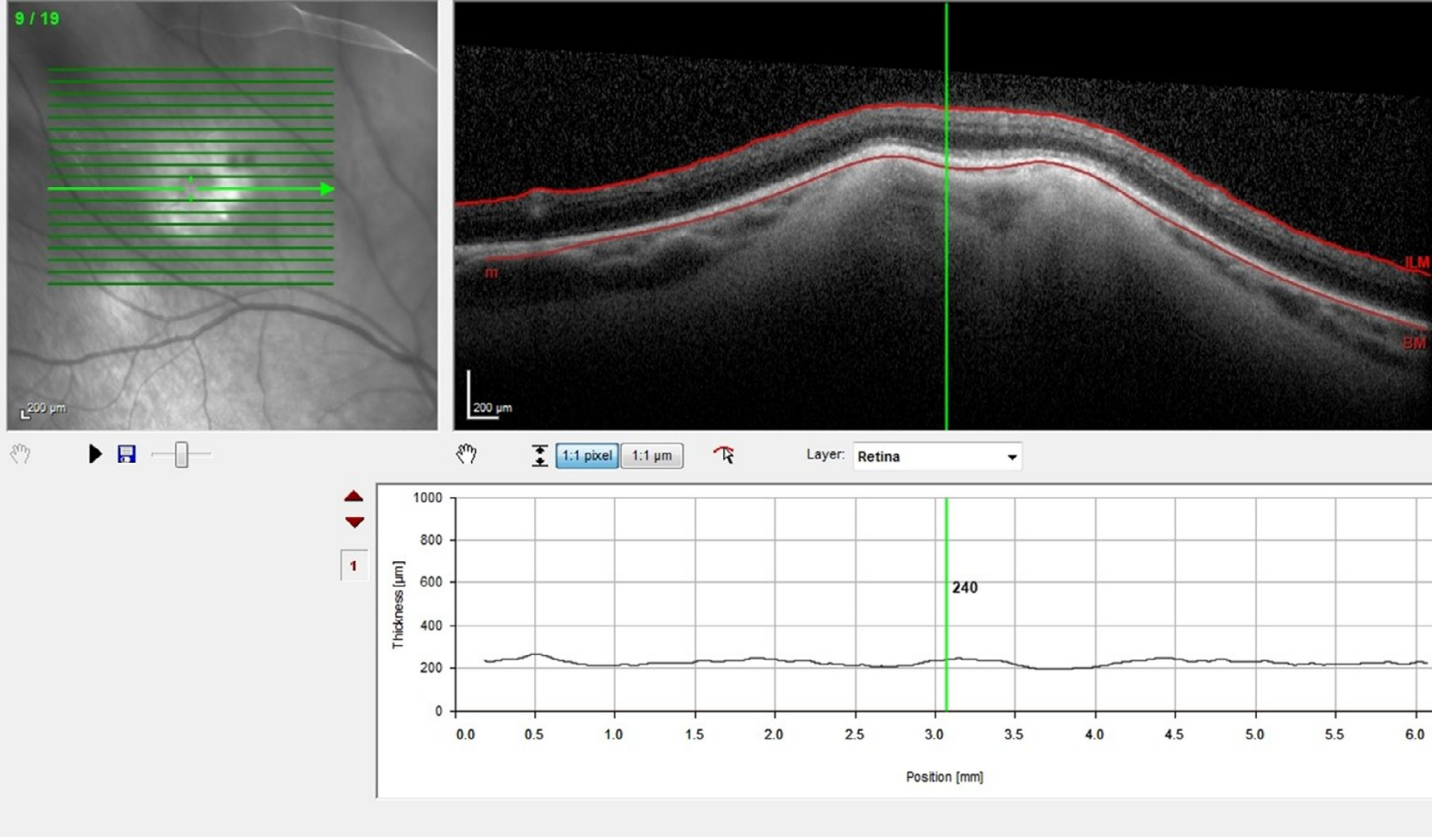
OCT through the right retinal lesion which shows a typical double "rocky and rolling" elevation with no subretinal fluid.

Enhanced depth optical coherence tomography (EDI-OCT) imaging through the right retinal lesion was obtained (Fig. 3) which shows a typical double “rocky and rolling” elevation with no sign of subretinal fluid or detachment.

## Outcome

A diagnosis of a right unilateral scleral choroidal calcification at the site of the superior oblique insertion was made with no globe injury related to the trauma. At maxillo-facial review, a decision was made not to repair the orbital fracture given the absence of diplopia and enophthalmos. The patient was discharged back to the care of their optometrist for regular review included dilated fundoscopy and EDI-OCT scans of the sclerochoroidal lesion.

## Discussion

This case highlights 2 main points: Ocular injuries and to a lesser extent retained intraocular foreign bodies, are extremely common in tripod facial fractures, requiring acute ophthalmology collaboration. Secondly, increasing the recognition of sclerochoroidal calcification as a masquerading condition to retained intraocular foreign body amongst other differential diagnoses.

Ocular injuries are common amongst facial fractures, highlighting the need for acute ophthalmology input. Al-Qurainy and colleagues [8] demonstrated that of the patients presenting over a 2-year period with a midfacial trauma sufficient to lead to a facial fracture, 90% sustained an ocular injury. Additionally, Septa and colleagues consistently revealed tripod fracture as the most common facial fracture sustained as a result of trauma, in particular most associated with road traffic accidents. Furthermore, of the total sustained tripod fractures, 84.5% resulted in ocular involvement, and 61% of such injuries were complicated by blindness or serious eye injury [9]. Due to the inherent traumatic nature of facial fractures, it is imperative that ophthalmology review is acutely obtained. As most patients presenting to a tertiary setting often have concurrent severe injuries and presentations, it is likely that radiological review will precede ophthalmology review. This case highlights the challenges that radiologists may encounter when other conditions masquerade as acute ocular injury in traumas that inherently have high ocular injury rates.

CT is the most practical step in assessing trauma cases as it is readily available and can detect not only osseous damage but a wide range of metallic intraocular foreign bodies safely at small limits of detection [10]. Most metals are unique and can be identified based on their core features, image artifacts, or degree of magnification and are usually described as hyper dense areas on CT [10]. This case demonstrates a hyper dense and highly attenuated lesion located within the posterior chamber of the eye, best observed in (Fig. 1). As intraocular foreign bodies are usually hyper dense and located in the posterior segment of the globe in most cases [4], it would be prudent to include retained intraocular foreign body as the top of the list of differential diagnoses.

The challenge arises when other conditions, such as sclerochoroidal calcification, masquerade as retained intraocular foreign bodies due to similar hyper dense appearances on CT imaging. Sclerochoroidal calcification is an idiopathic, usually benign, yet uncommon condition characterized by yellow-whitish subretinal lesions classically located in the superior temporal midperiphery of the fundus beyond the vascular arcades [11]. Despite its nomenclature, calcification is said to be contained within the sclera itself without extending into the choroid as new advancements in imaging modalities have shown [12]. Such advancements include enhanced depth imaging optical coherence tomography, or EDI-OCT, which allows for superior visualization of the sclera. Such scans demonstrated calcium deposits within the sclera only, exerting compressive forces on the choroid resulting in a “rocky” and “rolling” appearance on EDI-OCT [12]. Without the utilization of EDI – OCT sclerochoroidal calcification can clinically simulate several intraocular tumors such as choroidal metastasis, choroidal melanoma, or choroidal osteomas. Choroidal metastatic lesions present similarly to sclerochoroidal calcification lesions as they are both creamy-yellow in color. However, they are usually located within the vascular arcades specifically in close proximity to the macula and may present with a secondary subretinal detachment [13]. In cases of choroidal melanomas, they differ from calcification as they are usually pigmented, however in the case of amelanotic melanomas, they are distinct lesions with no underlying retinal pigmented epithelial dropout and are likely to induce a subretinal detachment. Choroidal osteomas also present similarly as they appear as distinct yellow-orange lesions, however they are often unilateral and confined closely to the optic nerve head. They also appear to have a predisposition for younger adults, specifically of the female sex. [13]. Additionally, sclerochoroidal calcifications usually manifest as multiple bilateral lesions beyond the vascular arcades, subsequently appearing as multiple hyper dense areas localized to the posterior segment on CT imaging.

In some rare cases, there may be systemic conditions associated with metastatic sclerochoroidal calcification usually involving abnormal calcium phosphorus metabolism or renal tubular hypokalemia metabolic alkalosis syndromes [14]. Patients with such conditions often present with systemic implications such as chronic muscle weakness, spasms, and episodic tetany which may warrant further investigation of calcium-phosphorus metabolism [14]. In general, sclerochoroidal calcification is an idiopathic condition that manifests as multiple yellow-white lesions scattered in the midperipheral fundus of asymptomatic older white individuals.

This case is a rare example of a singular unilateral lesion found in a uniquely young individual, under exceptional circumstances. Due to the complicated nature of this polytrauma scenario, specifically affecting the periorbital region, an initial diagnosis of intraocular foreign body was made based on CT findings. This case not only highlights this poorly understood entity, but it also demonstrates the significant power of interspecialty collaboration alongside multimodal imaging techniques on masquerading conditions.

## Summary

In summary, sclerochoroidal calcification is a rare and usually benign condition which may masquerade as a retained intraocular foreign body as in the case of this patient. As CT is usually the first imaging modality used in trauma cases, radiologists are among the first to assess scans and as such hyper dense lesions within the globe are more commonly thought to be as a result of retained intraocular foreign bodies, therefore adding complexity in differential diagnoses. Furthermore, this case highlights the benefits of multimodal imaging and likewise demonstrates a typically rare ocular condition of sclerochoroidal calcification.

## Patient consent

Written informed consent was obtained from the patient for the publication of this case report.
